# A novel lncRNA, RPL34-AS1, promotes proliferation and angiogenesis in glioma by regulating VEGFA

**DOI:** 10.7150/jca.59337

**Published:** 2021-08-27

**Authors:** Dongzhi Zhang, Haiping Jiang, Junyi Ye, Ming Gao, Xinzhuang Wang, Enzhou Lu, He Yang, Lixiang Wang, Shiguang Zhao

**Affiliations:** 1 Department of Neurosurgery, The First Affiliated Hospital of Harbin Medical University, Harbin, China.; 2 Key Colleges and Universities Laboratory of Neurosurgery in Heilongjiang Province, Harbin, China.; 3 Institute of Neuroscience, Sino-Russian Medical Research Center, Harbin Medical University, Harbin, China.; 4 Shenzhen University General Hospital, Xueyuan AVE 1098, Nanshan District, 11, Shenzhen, Guangdong, P. R. China.; 5 Department of Neurosurgery, The Affiliated Cancer Hospital of Harbin Medical University, Harbin, China.

**Keywords:** lncRNA, RPL34-AS1, glioma, proliferation, angiogenesis, VEGFA

## Abstract

**Purpose:** Brain gliomas are the most common primary malignant tumors of the central nervous system and one of the leading causes of death in patients with intracranial tumors. The lncRNA RPL34-AS1 is significantly upregulated in glioma tissues. However, the biological function of RPL34-AS1, especially in proliferation in glioma, remains unclear.

**Methods:** The role of RPL34-AS1 in proliferation and angiogenesis in glioma cells was investigated using the LN229, U87, and U251 glioma cell lines. The levels of RPL34-AS1 were detected using real-time quantitative reverse transcription polymerase chain reaction. CCK-8 and colony formation assays were performed to determine the role of RPL34-AS1 in proliferation and survival, and its role in angiogenesis was assessed by an endothelial tube formation assay. Changes in protein levels were assessed by western blotting.

**Results:** RPL34-AS1 was upregulated in glioma tissues and was correlated with tumor grade. RPL34-AS1 expression was also higher in glioma cells than in normal astrocytes. Knockdown of RPL34-AS1 blocked glioma cell proliferation by inhibiting angiogenesis. This effect occurred through decreased ERK/AKT signaling.

**Conclusions:** This study suggests that RPL34-AS1 affects cell proliferation and angiogenesis in glioma and therefore may potentially serve as a valuable diagnostic and prognostic biomarker and therapeutic target in patients with glioma.

## Introduction

Glioma is one of the most aggressive and common primary tumors of the central nervous system (CNS), accounting for almost 28% of all primary brain tumors and 80% of all malignant brain tumors 1. Glioma is divided into malignancy grades (WHO grade I to IV), which assists with determining the optimal treatment. Grades I and II indicate low-grade glioma (LGG), whereas grades III and IV are high-grade glioma (HGG) and have a worse prognosis 2. The prognosis for patients who are diagnosed with glioblastoma (WHO grade IV) is bleak, with a median survival time of less than 2 years 3. Surgery, chemotherapy, and radiotherapy remain the principal treatment modalities for glioma; however, only a small number of patients can achieve good results 4. Therefore, identifying the pathogenesis of glioma is of great significance for treatment.

Angiogenesis, or the growth of new blood vessels, is a key event that has been shown to be important in tumorigenesis and tumor progression 4. Angiogenesis plays crucial homeostatic roles, since blood vessels carry nutrients to tissues and organs and remove catabolic products. However, pathological angiogenesis can promote or facilitate numerous disease processes 5. As a high blood flow-dependent malignant tumor, glioma needs more support for tumor angiogenesis than other tumors 6. Gliomas stimulate the formation of new blood vessels through processes driven primarily by vascular endothelial growth factor A (VEGFA), which is the most important factor for promoting angiogenesis in tumors, including glioma 7,8. Studies have shown that VEGFA is upregulated in glioma 9 and is essential for the development of the disease 10. Therefore, an increasing number of studies have targeted tumor angiogenesis in the treatment of glioma.

LncRNAs are a type of noncoding RNA with a length >200 nucleotides with limited coding potential 11. LncRNAs are categorized according to their genomic location with respect to nearby protein-coding genes, namely, intergenic lncRNAs, intronic lncRNAs, and antisense lncRNAs 12. LncRNAs can regulate malignant behaviors in glioma, such as cell proliferation, metastasis, invasion, and drug resistance 13,14,15. Therefore, some lncRNAs have been considered as entry points for glioma treatment. For example, H19 is significantly overexpressed in CD133^+^ glioblastoma cells, which increases neurosphere formation in glioblastoma cells 16. HOTAIR is another important lncRNA overexpressed in glioma. HOTAIR can promote the malignant behavior of glioma through PI3K/AKT and MEK1/2 signaling by downregulating miR-326 16. Ribosomal protein L34 antisense RNA 1 (RPL34-AS1) is a novel lncRNA that inhibits invasion in papillary thyroid cancer cell lines 17 and inhibits esophageal cancer cell proliferation by downregulating RPL34 expression 18. The same effect has been observed in colorectal and gastric cancers 19,20. We found increased expression of RPL34-AS1 in glioma; however, its biological effect on glioma remains unknown.

## Materials and Methods

### Human glioma specimens

Human glioma specimens were obtained from patients undergoing initial surgery who were diagnosed with glioma at the Department of Neurosurgery, The First Affiliated Hospital of Harbin Medical University and Department of Neurosurgery, The Affiliated Cancer Hospital of Harbin Medical University between September 2019 and December 2020. The grading of glioma specimens was determined by a neuropathologist according to the 2007 WHO classification. All patients signed an informed consent. The samples were stored at -80°C before being embedded in paraffin. Normal brain tissues were obtained from patients with severe traumatic brain injury who underwent decompression surgery and were used as negative control. The study was approved by the Ethics Committee of The First Affiliated Hospital of Harbin Medical University.

### Cell lines and culture conditions

Human glioma cell lines (A172, U87, U251, LN229 and T98G), normal human astrocytes (NHA), and human umbilical vein endothelia cells (HUVECs) were obtained from the China Infrastructure of Cell Line Resource (National Science & Technology Infrastructure, NSTI). All cell lines were recently authenticated by STR analysis and tested for mycoplasma contamination. Glioma cell lines and NHA were cultured with Dulbecco's Modified Eagle's Medium (DMEM) containing 10% fetal bovine serum (FBS). HUVECs were cultured in High-glucose Dulbecco's modified Eagle's medium (DMEM) and DMEM-F12 mixed medium containing 10% FBS. Routine culture was performed in a humidified incubator maintained at 37 °C with 5% CO_2_ and 95% air.

### Cell transfection

The Lipofectamine 2000 reagent was used to transfect the glioma cell lines with siRNAs according to the manufacturer's instructions. The siRNAs were purchased from GenePharma (Shanghai, China).

### Quantitative real-time PCR (qRT-PCR)

Total RNA was extracted using TRIzol reagent (Invitrogen, USA). The RNA concentration and quality were determined at 260/280 nm ratio using a NanoDrop spectrophotometer (Thermo Scientific™ NanoDrop 2000c). Reverse transcription was performed using a PrimeScript RT reagent kit (ToYoBo). A SYBR Green PCR Master Mix kit (Roche, Germany) was utilized to verify the expression of the target gene. qRT-PCR was performed using an ABI 7500 Real-Time PCR. Relative expression was normalized to that of endogenous controls using the comparative cycle threshold method, and the fold change in gene expression was calculated using the 2^-∆∆Ct^ method. RPL34-AS1-f: AAAGAGCAAAGGCTGCTCAC, RPL34-AS1-r: TGATGGCTTCTTCAACCAGGA.

### Cell viability assay

U87, U251, and LN229 cells were treated with siRNAs and then seeded (4000 cells/well) in 96-well plates the next day. Cell proliferation was measured through the CCK-8 assay. After the addition of 10 μL of CCK-8 solution, the cells were incubated at 37 °C for one hour. Absorbance at 450 nm was measured with a spectrophotometer. The experiment was repeated at least three times.

### Colony formation assay

U87, U251, and LN229 cells (1000 cells/well) were seeded into 6-well plates and cultured for 10 days. The colonies were fixated in 4% formaldehyde for 30 minutes and then stained with 0.1% crystal violet for ten minutes. Viable colonies that contained more than 50 cells were counted. The experiment was performed at least thrice for each cell line.

### Flow cytometric analysis of cell apoptosis

The FITC Annexin V Apoptosis Detection Kit was used to stain the glioma cell lines according to the manufacturer^'^s recommendations. Cell apoptosis was then measured by a FACScan flow cytometry.

### Western blot analysis

Protein samples were extracted from the glioma cell lines by RIPA buffer, which contains phenylmethylsulfonyl fluoride (PMSF) and a phosphatase inhibitor. Protein samples were separated by 7-12.5% SDS-PAGE gels and transferred to polyvinylidene difluoride (PVDF) membranes. The membranes were blocked with 5% skim milk and incubated with the primary antibody against VEGFA, actin, Akt, p-Akt, Erk, p-Erk, BCL-2, and BAX at 4 °C overnight. The membranes were incubated with fluorescent-dye conjugated secondary antibodies at room temperature for two hours the next day. The membranes were observed using a ChemiDoc XRS + Imaging System.

### Tube formation assay

A standard Matrigel assay was performed to evaluate *in vitro* angiogenesis activity by quantifying the tube formation. The 96-well culture plates were coated with 80 μl of Matrigel per well, and then allowed to polymerize for thirty minutes at 37 °C. HUVECs (1.5×10^4^ cells/well) were grown in the absence or presence of 100% conditional medium that the supernatant of the cells was collected and centrifuged at 2,000×g for 20 minutes at 4 °C at 48 h after the transfection for 6 h at 37 °C in a 96-well plate. Each well was photographed at ×100 magnification using microscopy. The total tubule length and number of tubule branches were measured through Image J software.

### Statistical analysis

Statistical analyses comparing data between groups were performed using Student's t-test or one-way ANOVA. The association between target gene and clinical features was analyzed using Pearson's chi‐square test. Data are presented as mean ± standard error of the mean (SEM). P<0.05(*) was considered to indicate a statistically significant result. All statistical analyses were carried out using (Prism software version 7.0).

## Results

### RPL34-AS1 is upregulated in glioma tissues and cell lines, and its expression is correlated with tumor grade

**Figure 1:**
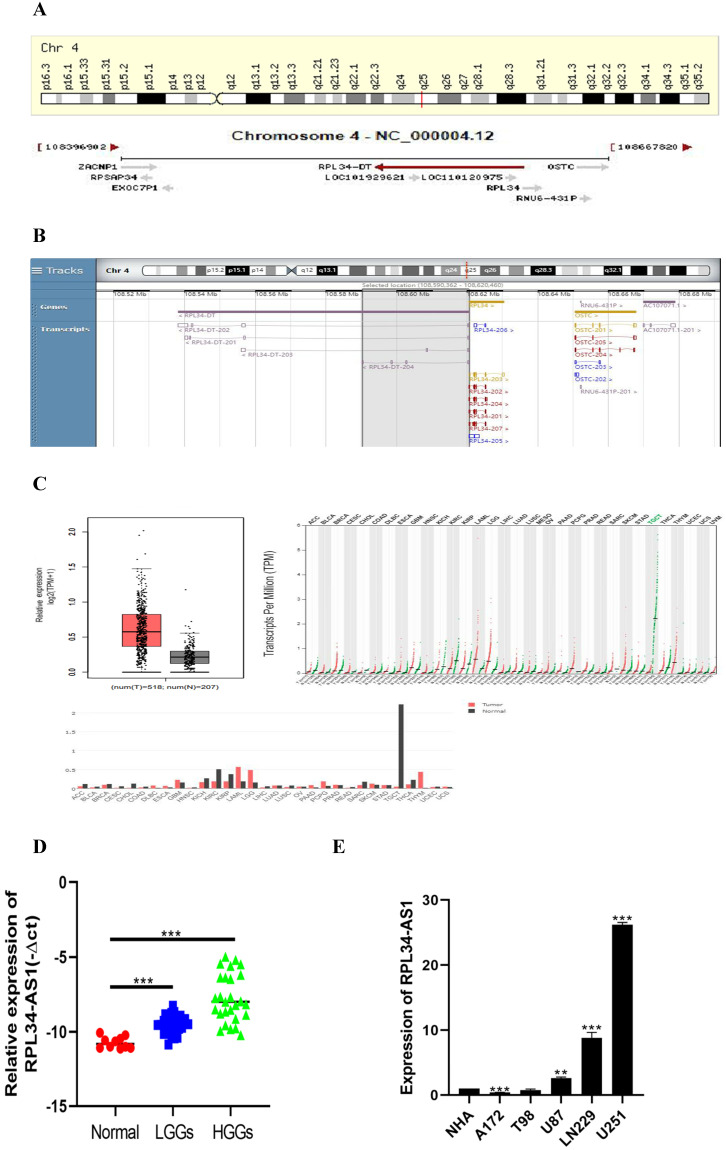
** RPL34-AS1 is upregulated in glioma. (A, B)** RPL34-AS1 is localized at the human chromosome 4q25 and highly conserved in human. **(C)** The gene expression profile across all tumor samples and paired normal tissues. RPL34-AS1 is upregulated in glioma tissues compared with normal brain tissues from TCGA and GTEx. **(D)** The differential expression of RPL34-AS1 in glioma specimens of different grades and normal brain tissue specimens. **(E)** The expression of RPL34-AS1 examined by qPCR in five glioma cell lines and normal human astrocytes cell line (NHA). *P < 0.05; **P<0.01; ***P<0.001.

To better understand its role in glioma, we performed analyses from GENECARDS and RNAcentral that RPL34-AS1 is localized to human chromosome 4q25 (Figure 1A) and highly conserved in humans (Figure 1B). The expression of RPL34-AS1 was higher in 518 glioma tissues than in 207 normal tissues (Figure 1C), according to GEPIA data from TCGA and GTEx. We then performed qRT-PCR to verify the expression of RPL34-AS1 in glioma tissues and normal brain tissues. We found high RPL34-AS1 expression in glioma tissue specimens, especially in HGGs, compared to that in normal brain tissue specimens (Figure 1D).

We divided the 56 patients with glioma into a high RPL34-AS1 expression group (n = 34) and a low expression group (n = 22) (Table 1) and analyzed the correlation between RPL34-AS1 expression and clinicopathological features. As shown in Table 1, high RPL34-AS1 was significantly correlated with tumor grade (P = 0.029) but had no significant correlation with age, sex, or KPS (P > 0.05).

Moreover, the expression of RPL34-AS1 in glioma cell lines and normal human astrocytes (NHAs) was also evaluated. We observed notably high expression of RPL34-AS1 in LN229, U87, and U251 cells compared with the expression in NHAs (Figure 1E). Because the expression of RPL34-AS1 was highest in these glioma cell lines, we used LN229, U87, and U251 cells for further experiments.

### Knockdown of RPL34-AS1 suppresses proliferation in GBM cell lines

**Figure 2:**
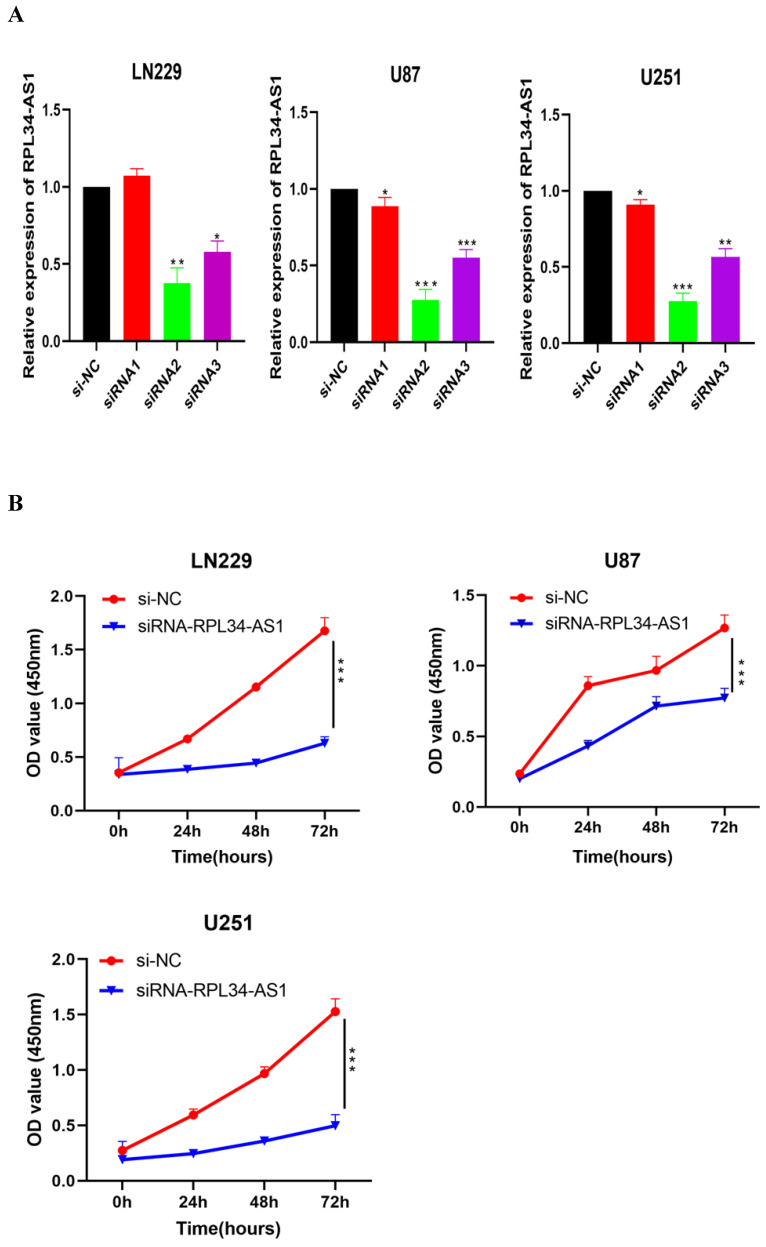

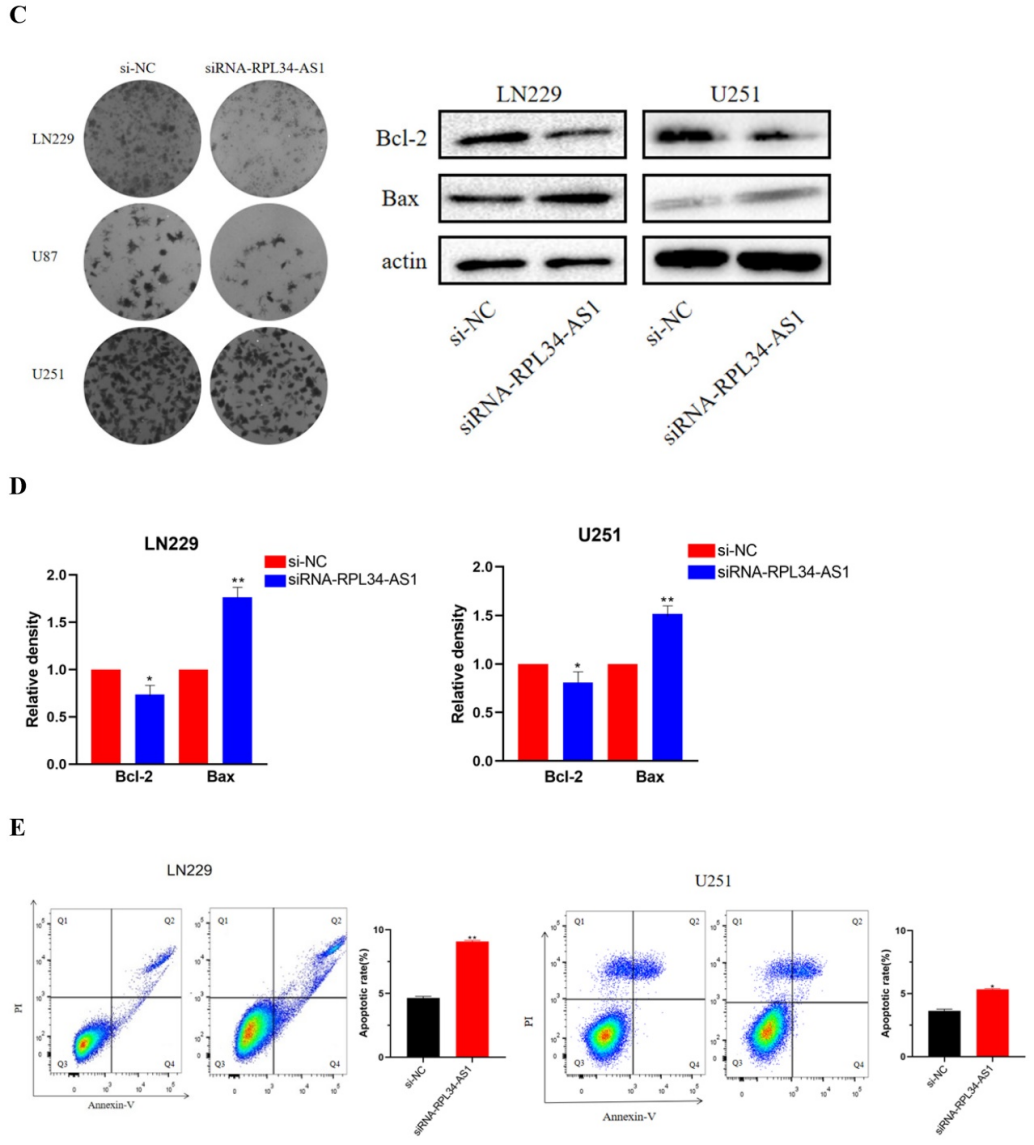
** Knockdown of RPL34-AS1 inhibits proliferation in glioma cell lines. (A)** Decreased RPL34-AS1 level in LN229, U87 and U251 cell lines transfected with RPL34-AS1 siRNAs compared with siRNA-NC. **(B)** Knockdown of RPL34-AS1 impaired the proliferation of glioma cells compared with group of si-NC. **(C)** siRNA-RPL34-AS1 inhibit colony formation of the glioma cell lines. **(D)** Western blot analysis revealed that siRNA-RPL34-AS1 increased Bax expression and decreased Bcl-2 expression. **(E)** Flow cytometric analysis also showed that silencing of RPL34-AS1 induced apoptosis in glioma cell lines *P < 0.05; **P<0.01; ***P<0.001.

To explore the functional relevance of RPL34-AS1 in glioma cells, we interfered with its expression by transfecting cells with RPL34-AS1 siRNAs. We validated the knockdown of RPL34-AS1 by qRT-PCR (Figure 2A). CCK-8 assays showed that knockdown of RPL34-AS1 impaired the proliferation of glioma cells (Figure 2B). We performed a colony formation assay to assess the effect of RPL34-AS1 on glioma cell survival (Figure 2C). We also tested the expression of the apoptosis-related factors Bcl-2 and Bax by western blot after knockdown of RPL34-AS1 (Figure 2D). Flow cytometry assays also revealed that silencing RPL34-AS1 induced apoptosis in LN229 and U251 glioma cells (Figure 2E). These data suggest an oncogenic role of RPL34-AS1 in glioma progression.

### RPL34-AS1 promotes endothelial cell angiogenesis by regulating VEGFA

**Figure 3:**
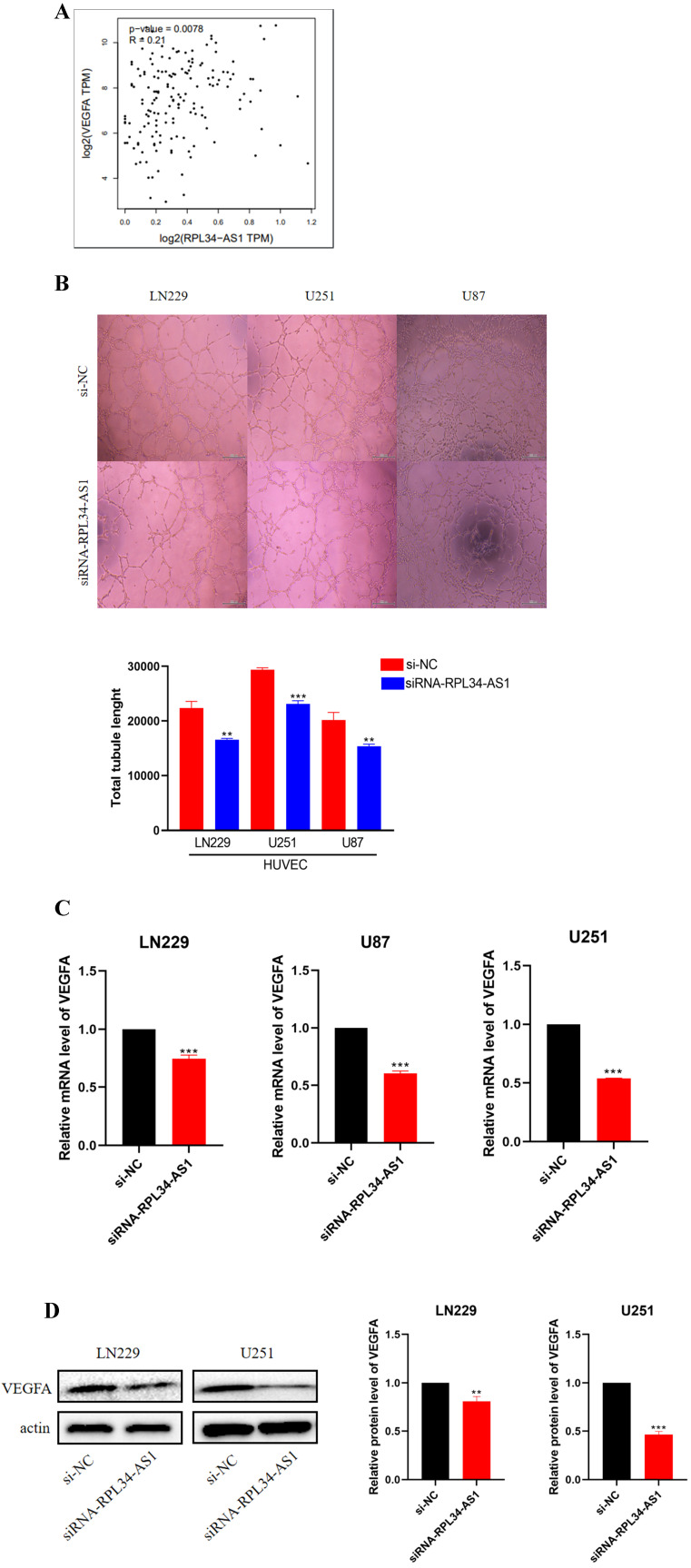
** RPL34-AS1 promotes endothelial cell angiogenesis by regulating VEGFA. (A)** Biological analysis confirmed the positive correlation between RPL34-AS1 and VEGFA. **(B)** A tube formation assay was used to analyse the impact of RPL34-AS1 on HUVECs. **(C, D)** qRT-PCR and Western blotting assay were performed to detect the expression of VEGFA. *P < 0.05; **P<0.01; ***P<0.001.

Glioma cells can produce a large number of proangiogenic factors to stimulate angiogenesis, which helps to supply oxygen and nutrients to highly proliferative tumor cells 21. Several clinical trials of antiangiogenic drugs in patients with newly diagnosed or recurrent gliomas have supported this view 22. We conducted a biological analysis, which confirmed a positive correlation between RPL34-AS1 and VEGFA (Figure 3A). Therefore, we examined whether RPL34-AS1 has an effect on angiogenesis in glioma using a tube formation assay. The effect of RPL34-AS1 knockdown on HUVECs was assessed by relative tube length. The conditioned media from si-RPL34-AS1 glioma cells exhibited a strong negative effect on HUVEC tube formation compared with that from si-NC cells (Figure 3B). We then measured the expression of VEGFA at the mRNA (Figure 3C) and protein levels (Figure 3D) using qRT-PCR and western blot, respectively.

### Knockdown of RPL34-AS1 suppresses VEGFA production via regulation of ERK/AKT signaling

**Figure 4:**
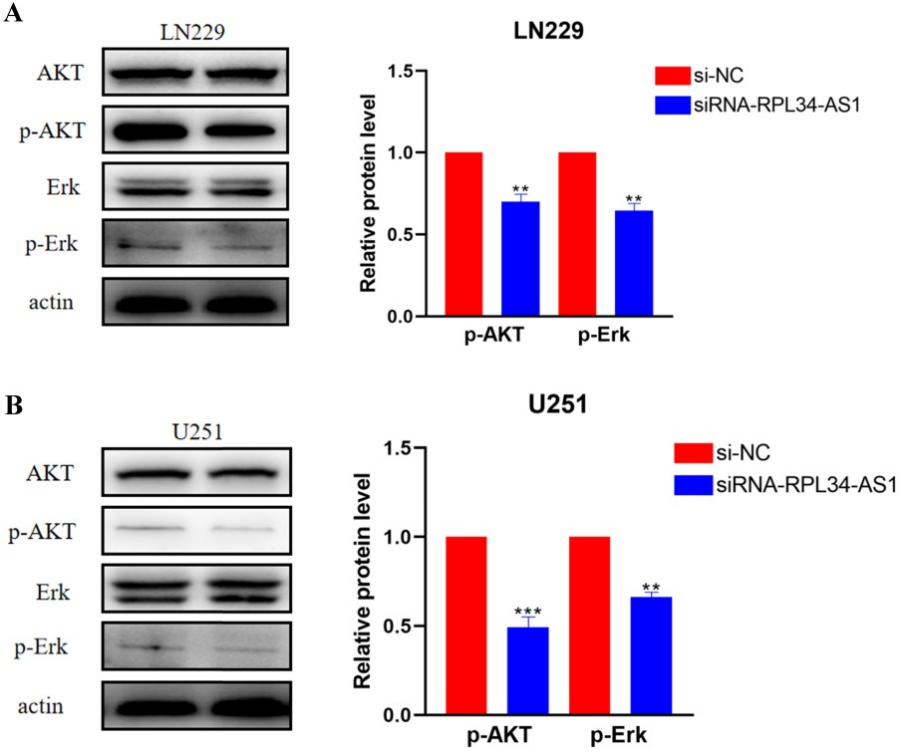
** RPL34-AS1 exerts tumour promoter function through the Erk/AKT signaling. (A, B)** Western blotting assay were performed to detect the expression on relative protein level of Erk/AKT signaling. *P < 0.05; **P<0.01; ***P<0.001.

Activation of ERK/AKT signaling in glioma cells plays an important role in indirectly activating VEGFA, which plays a crucial role in tumor angiogenesis 23,24 through the regulation of various endothelial functions 25,26,27. To confirm whether the contribution of RPL34-AS1 to glioma development was mediated through the ERK/AKT signaling pathway, we assessed the expression of ERK, AKT, p-ERK, and p-AKT by western blot (Figure 4A, 4B). The expressions of p-ERK and p-AKT were significantly decreased in cells transfected with si-RPL34-AS1 compared to cells transfected with si-NC, whereas there were no obvious changes observed in the expressions of total ERK and AKT.

## Discussion

In this study, we report for the first time that the lncRNA RPL34-AS1 is highly expressed in gliomas, especially in HGGs. The expression of RPL34-AS1 was associated with poor prognosis and was related to glioma cell proliferation *in vitro*. We also proved that RPL34-AS1 mediates angiogenesis in glioma by regulating VEGFA. This study indicates that RPL34-AS1 may be a potential target for the treatment of glioma.

Glioma is the most common primary intracranial tumor and is derived from the carcinogenesis of glial cells in the neuroectoderm. Glioma accounts for 80% of primary intracranial malignant tumors 2. It is characterized by excessive proliferation, recurrence, and high mortality. Surgery is currently the most important treatment for glioma, and with the rapid development of brain functional imaging, neuronavigation, electrophysiology, and other technologies, the maximum range of safe resection of tumors has become attainable. However, postoperative survival is not promising due to the biological characteristics of glioma. Because of the invasive nature of glioma, especially GBM, the median survival period is only 14.6 months, and the survival rate at 5 years after diagnosis is only 5.8% 3. Therefore, it is necessary to study the pathogenesis of glioma at the molecular level to improve treatment options.

An increasing number of studies have reported that lncRNAs demonstrate remarkable functions in different tumors in terms of gene expression, mRNA regulation, protein modulation, and epigenetic regulation. Moreover, aberrant expression of lncRNAs in various human tumors has been demonstrated in recent years. There are significantly different lncRNA expression profiles in glioma tissue and normal brain tissue, and these differentially expressed RNAs are involved in glioma progression, complex conditions, and poor prognosis 21,28,29. Here, we demonstrated that RPL34-AS1 expression was increased in glioma cell lines and glioma tissues, and RPL34-AS1 was significantly associated with WHO grade. Moreover, lncRNA RPL34-AS1 had a significant influence on cell proliferation and apoptosis, and silencing RPL34-AS1 reduced the proliferation of glioma cells *in vitro*. However, the mechanism by which it acts as an oncogene in glioma was still unknown.

**Figure 5:**
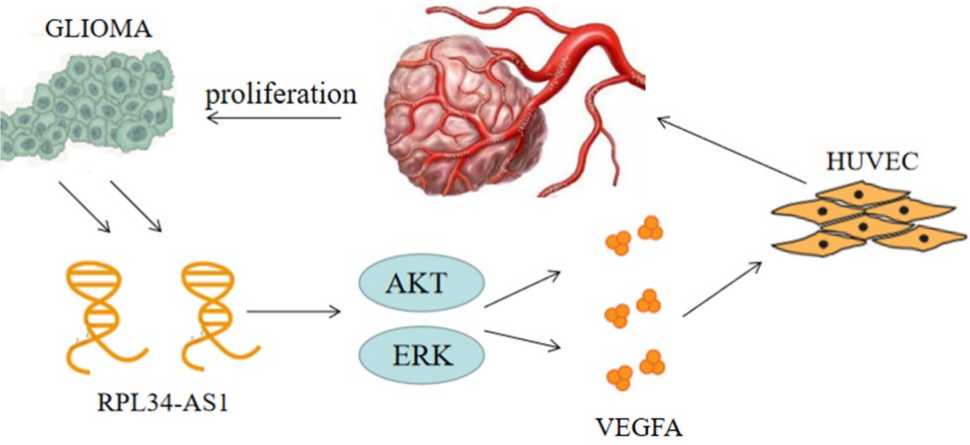
The schematic representation of the mechanism on lncRNA RPL34-AS1 to ErK/AKT/VEGFA axis in glioma proliferation and angiogenesis.

Continuous angiogenesis has become the focus of our research as one of the biological hallmarks of tumors (Figure 5). It is well known that VEGFA is one of the most important factors in glioma angiogenesis. VEGFA has the capacity to induce physiological and pathological angiogenesis 30 to meet the nutrient needs of proliferating tumors. Glioma cells regulate angiogenesis through the angiogenic cytokine VEGFA, which signals to vascular endothelial cells in the tumor microenvironment 21. This is a pathological characteristic of HGG. However, whether VEGFA is regulated by the lncRNA RPL34-AS1 remains largely unknown. Our biological analysis showed a positive correlation between RPL34-AS1 and VEGFA, suggesting that RPL34-AS1 may play a role in regulating VEGFA. Knockdown of RPL34-AS1 significantly decreased expression of VEGFA at the mRNA and protein levels, as well as tube formation of endothelial cells. Therefore, we conclude that RPL34-AS1 plays an important pathological role in glioma by affecting tumor angiogenesis.

Numerous signaling pathways have been implicated in VEGFA-induced endothelial cell proliferation, typically AKT and ERK, which activate glioma cells and indirectly activate VEGFA through their action on HIF-1α 31,32. Previous studies have found that when the ERK/AKT pathway is active, it can effectively promote the proliferation and invasion of tumor cells, and this process is closely related to VEGFA 33. ERK signaling is the main pathway involved in signal transduction in vascular endothelial cells 34. In addition, the AKT signaling pathway, which can promote the transcription of VEGFA, is widely active in brain tissue. This transcription stimulates the production of VEGFA to promote angiogenesis 35. Furthermore, we have previously shown that silencing RPL34-AS1 led to a significant change in EGFR expression. As the upstream gene of the ERK/AKT pathway, EGFR is closely related to the proliferation, invasion, and migration of tumor cells 36. The specific mechanism will be the aim of our future research.

In conclusion, our study showed that RPL34-AS1 expression is upregulated in glioma tissues and cell lines. Knockdown of RPL34-AS1 suppresses proliferation and induces apoptosis in glioma cell lines. RPL34-AS1 exerts oncogenic activity partially by affecting angiogenesis in glioma by regulating the VEGFA and ERK/Akt signaling pathways. Therefore, RPL34-AS1 may be a potential novel target for glioma therapy.

## Figures and Tables

**Table 1:**
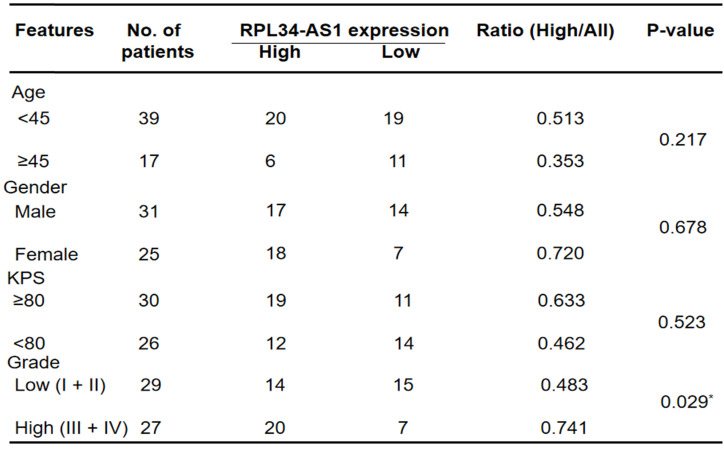
Correlation between lncRNA RPL34-AS1 expression level and clinicopathological characteristics in glioma patients (n=56)

## Abbreviations

LGG: low-grade glioma
HGG: high-grade glioma
VEGFA: vascular endothelial growth factor A
lncRNA: Long non-coding RNA
PI3K: phosphoinositide 3-kinase
AKT: Serine/threonine-protein kinase
ERK: extracellular regulated protein kinases
RPL34-AS1: ribosomal protein L34 antisense RNA 1
RPL34: ribosomal protein L34
NHAs: normal human astrocytes
GBM: glioblastoma multiforme
HUVECs: human umbilical vein endothelial cells
HIF-1α: hypoxia inducible factor-1α
EGFR: epidermal growth factor receptor
